# Seven decades of nontuberculous mycobacteria in Denmark: shifts in species distribution and clinical relevance

**DOI:** 10.1128/jcm.01561-25

**Published:** 2026-04-20

**Authors:** Xenia Emilie Sinding Iversen, Anders Norman, Dorte Bek Folkvardsen, Erik Michael Rasmussen, Erik Svensson, Leen Rigouts, Conor Meehan, Lars Jelsbak, Troels Lillebaek

**Affiliations:** 1 International Reference Laboratory of Mycobacteriology, Statens Serum Institut 4326, Copenhagen, Denmark; 2 Mycobacteriology/Institute of Tropical Medicine 37463 , Antwerp, Belgium; 3 Department of Biosciences, Nottingham Trent University 6122, Nottingham, United Kingdom; 4 Department of Biotechnology and Biomedicine, Technical University of Denmark 158981, Kgs. Lyngby, Denmark; 5 Global Health Section, Department of Public Health, University of Copenhagen 86986, Copenhagen, Denmark; University of Western Australia, Perth, Western Australia, Australia

**Keywords:** nontuberculous mycobacteria, clinical, occurrence, species distribution, pathogen evolution

## Abstract

The global burden of human disease caused by nontuberculous mycobacteria (NTM) is steadily increasing. Using our unique historical collection of freeze-dried mycobacterial isolates at the Statens Serum Institut, Copenhagen, we investigated long-term trends in NTM clinical relevance and species distribution in Denmark over the past seven decades. A total of 666 historical NTM isolates from human samples collected between 1948 and 1978 were compared with 1,820 contemporary isolates identified during routine diagnostics from 2013 to 2022. Species identification was performed for all isolates using the GenoType CM and AS line-probe assays. Among historical isolates, 16 different species were identified, with the majority classified as *Mycobacterium* sp. (32.1%, *n* = 214). In contrast, recent isolates were dominated by *Mycobacterium avium* (39.6%, *n* = 721), while *Mycobacterium* sp. accounted for only 7.8% (*n* = 142). Furthermore, notable changes in prevalence over time at the single species level were observed for *M. avium* and *Mycobacterium abscessus*. Further genomic analyses of historical *Mycobacterium* sp. isolates revealed several uncommon or previously unrecognized species rarely detected in current diagnostic settings. In conclusion, our findings suggest a significant shift in the spectrum of species responsible for human NTM infections in Denmark over the past seven decades, with possible implications for diagnostic strategies, clinical management, and public health surveillance.

**IMPORTANCE:**

Infections caused by nontuberculous mycobacteria (NTM) are increasingly recognized worldwide, yet the long-term epidemiology of these human pathogens remains poorly understood. By leveraging a unique historical collection of mycobacterial isolates, this study suggests a shift in the species spectrum responsible for NTM infections in Denmark over the past seven decades, with *Mycobacterium avium* now predominating. Sequencing of historical isolates further uncovered several rare or previously unrecognized *Mycobacterium* species, illustrating the dynamic nature of NTM epidemiology over time. These findings underscore the need for sustained surveillance, precise species-level identification, and heightened clinical awareness to guide effective management of NTM infections amid a continually evolving disease landscape.

## INTRODUCTION

Nontuberculous mycobacteria (NTM) belong to the genus *Mycobacterium*, excluding the *Mycobacterium tuberculosis* complex (MTBC), causing tuberculosis (TB), and *Mycobacterium leprae* and *Mycobacterium lepromatosis*, causing leprosy (1, 2). NTM is a ubiquitous environmental organism commonly found in water or soil. More than 200 species have been identified to date, although only a fraction are known to cause disease in humans (3).

The first reports of NTM disease in humans date back to the 1930s (4). However, their clinical importance became more widely recognized in the 1950s and 1960s, as increasing numbers of acid-fast bacilli (AFB), distinct from MTBC, were successfully identified in human samples (4, 5). This growing awareness prompted efforts to classify these organisms, initially based on growth rate, dividing them into rapidly growing mycobacteria (RGM) and slowly growing mycobacteria (SGM). In 1959, the Runyon classification system further categorized the SGM based on pigment production: Runyon I (photochromogens, producing yellow-orange pigment upon exposure to light), Runyon II (scotochromogens, producing pigment regardless of light), and Runyon III (nonchromogens, lacking pigment production). RGM were classified as Runyon IV, and most appeared nonchromogenic. The distinction between rapid and slow growth remains widely used today (5–7).

As awareness of NTM infections has grown in recent decades, various diagnostic methods have been developed for accurate species identification (8). Early diagnostic approaches relied on AFB staining and microscopy, time-consuming biochemical tests, and phenotypic characterization of isolates cultured on egg-based media (9, 10). Importantly, species-level identification of early isolates was constrained not only by methodological limitations but also by the limited number of NTM species described at the time. In the late 1900s, molecular methods such as nucleic acid hybridization, high-performance liquid chromatography, and later Sanger DNA sequencing were introduced into routine diagnostics (11, 12). These techniques have since been refined with improved culture media, probe hybridization assays, and more recently, single- and multi-locus DNA sequencing, which now play a central role in species- and subspecies-level identification. These diagnostic improvements have led to greater recognition of the clinical relevance of NTM and have contributed to the observed global increase in NTM infections and disease incidence (13, 14). Nevertheless, challenges persist, especially due to limited long-term epidemiological data on NTM occurrence and etiology.

For decades, NTM have been of diagnostic and scientific interest at the International Reference Laboratory of Mycobacteriology (IRLM) at Statens Serum Institut (SSI). The SSI has been the central unit for mycobacterial diagnostics and surveillance in Denmark since 1910, enabling the systematic collection of selected clinical isolates and associated metadata from patients with suspected mycobacterial infection since 1948.

In this study, we investigate trends in the clinical occurrence and species distribution of NTM in humans in Denmark over the past seven decades using both historical and contemporary isolates. For that purpose, we use selected strains from the unique strain collection at IRLM, which includes isolates from at least a decade before the widespread use of antibiotics, providing an unparalleled opportunity to study the long-term epidemiology of NTM in Denmark.

## MATERIALS AND METHODS

### Isolate selection and preparation

Isolates were selected from the IRLM strain collection through a retrospective review of historical strain records based on the following inclusion criteria: (i) isolates were obtained from humans between 1948 and 1978; (ii) they had a historical species designation compatible with NTM (e.g., saprophyte); and (iii) associated metadata were available, including sample material, applicant information, year of isolation, and geographical origin in Denmark (see flowchart in Fig. S1). The clinical specimen type from which the NTM was isolated was not a criterion for inclusion or exclusion. Among the historical isolates, 24 fulfilling the inclusion criteria were previously examined using whole-genome sequencing (WGS) and included in the study (15).

For each freeze-dried isolate, bacterial cells were suspended in 3 mL Dubos medium (SSI Diagnostica, Hillerød, Denmark) and split into two parts: for initial genotypic species identification and for subsequent analyses. All experimental procedures were conducted in a biosafety level 3 laboratory, where the historical strain collection is stored.

### Preliminary species determination using line-probe assays

DNA for the line-probe assay (LPA) analyses was extracted directly from freeze-dried cell suspensions using the FluoroLyse kit (v1.0), according to the manufacturer’s instructions for cultures (Hain Lifescience GmbH, Nehren, Germany). Species identification was performed for all isolates using the GenoType Mycobacterium CM (v2.0, Hain Lifescience) to obtain an initial species-level classification (see LPA classifications in Fig. S2). Further identification was conducted using the GenoType Mycobacterium AS assay (v1.0, Hain Lifescience) exclusively for isolates classified by GenoType CM as *Mycobacterium malmoense* or *Mycobacterium marinum*/*Mycobacterium ulcerans*, in accordance with the manufacturer’s recommendations. Additional testing using the GenoType NTM-DR (v1.0, Hain Lifescience) was performed for isolates identified as *Mycobacterium intracellulare* and *Mycobacterium abscessus* to identify on subspecies level. All LPAs were performed and interpreted following the manufacturer’s instructions. These methods are ISO 17025 accredited and tested through EQA panels.

### NTM species distribution of historical versus contemporary isolates

To compare the NTM species distribution between historical and contemporary isolates, we collected LPA data on isolates received at the IRLM, SSI, from 2013 to 2022. The percentage distribution of single species identified by the GenoType CM assay was calculated for both data sets and visualized using Microsoft Excel 2019. Statistical significance of observed differences was calculated using Fisher’s exact test in R (v4.4.0).

The 24 historical isolates from a previous study were identified by WGS, based on average nucleotide identity (ANI) distances to NCBI RefSeq type strains instead of the GenoType CM assay (15). GenoType CM species classifications were inferred from the closest ANI-determined species for these isolates, as such testing had not been performed previously. Isolates identified as species not included in the GenoType CM assay were categorized as *Mycobacterium* sp. for comparison purposes.

### DNA extraction and library preparation for whole-genome sequencing

A subset of isolates characterized as *Mycobacterium* sp. (uncharacterizable mycobacterial species detected by GenoType CM, *n* = 30) and *Mycobacterium gordonae* (*n* = 5), as well as isolates showing invalid band patterns (designated as “unknown,” *n* = 5), was subjected to WGS. The 30 isolates classified as *Mycobacterium* sp. were selected by randomly choosing one to four isolates from each year in which *Mycobacterium* sp. isolates were represented. All of these isolates originated from pulmonary clinical samples. DNA was extracted following the instructions of the QIAmp DNA Mini Kit (Qiagen, Hilden, Germany), with minor modifications as previously described (15). DNA concentrations were measured using the Qubit dsDNA HS Assay kit (range: 0.1–120 ng) and the Qubit 2.0 Fluorometer, following the manufacturer’s instructions (Life Technologies, Thermo Fisher Scientific Inc., Waltham, MA, USA), to achieve a final input concentration of 0.2–0.5 ng/mL.

Sequencing libraries were prepared using the Nextera XT DNA Preparation Kit (Illumina, San Diego, CA, USA), following the manufacturer’s instructions with quantitative normalization of library concentrations. Libraries were sequenced using 300 cycles of paired-end sequencing and the MiSeq Reagent Kit (v2) on the Illumina MiSeq sequencing platform (Illumina, 2 × 150 bp read length).

### Whole-genome sequencing data quality checks and *de novo* assembly

All paired-end sequence libraries were prepared for bioinformatic analysis using the FASTQ-preprocessor tool fastp (0.23.2), which removes sequencing adapter fragments, trims low-quality ends, and merges and error-corrects overlapping paired reads. Overall sequence quality, presence of contaminating DNA fragments, estimated genome size, and coverage were assessed using the following suite of software tools: the SeqKit Stats Module (v2.4.0), the kmer-counting tool KMC (v2.3.1), and the taxonomic sequence classifier Kraken 2 (v2.1.2), in combination with the Bayesian species abundance tool Bracken (v2.5). These tools were applied against a compact (12 GB) custom database containing all known mycobacterial species and a selection of commonly seen contaminating bacteria, archaea, eukaryotes (*Homo sapiens* and fungi), and viruses. Passing criteria for paired-end libraries were a sequencing error rate of <1.0%, an estimated genome size between 3.0 and 8.0 Mbp, and genome coverage of >30×, all estimated from kmer frequencies calculated with KMC. Additionally, any sample with less than 98% of Kraken-classified reads assigned to mycobacteria was discarded or re-cultured for renewed WGS. *De novo* assemblies were constructed using the Shovill Assembly Pipeline by Torsten Seemann (v1.1.0, https://github.com/tseemann/shovill) with the following optional parameters: --minlen 300—mincov 5—trim. The SeqKit Stats Module was used to extract the assembly contig length metrics, such as N50, and the tool CheckM (v1.2.2) was used to assess genome completeness and to verify the absence of contaminants.

### *In silico* species identification of mycobacteria

Species identification of samples was performed in two steps: first, by running the kmer-based NTM-profiler tool by Jody Phelan (v0.3.0, https://github.com/jodyphelan/NTM-Profiler) on raw sequencing reads, which identifies most known species of NTM with an ANI ≥95%, including samples containing mixtures of mycobacterial species; and second, for samples deemed to contain single species, we calculated ANI of the *de novo* assembled contigs against a database containing all known assembled genomes of representative species and subspecies of mycobacteria, downloaded from NCBI on 19 June 2023 using FastANI (v1.32).

### Core genome phylogeny based on pangenome-derived genome annotation

We used the bacterial genome annotation and pan-genome analysis tool ggCaller (v1.3.6) on all samples belonging to the genus *Mycobacterium*, as well as any representative genome that fell within an ANI of 85% of any of the included samples. We ran ggCaller with the following optional parameters: --annotation ultrasensitive --aligner def --alignment core --core-threshold 0.8 --identity-cutoff 0.8 --len-diff-cutoff 0.9 --family-threshold 0.7 --merge-paralogs --clean-mode strict. The resulting core gene alignment, consisting of 1,428 concatenated single-copy genes, was analyzed with IQ-TREE (v1.6.2) using the following parameters to construct a maximum-likelihood phylogeny: -alrt 1000 -bb 1000 -mset GTR. IQ-TREE identified the model GTR + F + R10 as the highest-scoring model using the Bayesian information criterion. The isolate Mu0851, identified as *Mycobacterium chelonae* subsp. *gwanakae*, had to be excluded from the main core gene phylogeny, as ggCaller kept failing when attempting to include the more distantly related Abscessus-Chelonae clade into the analysis (see separate phylogeny of the Abscessus-Chelonae clade in Fig. S3). We therefore ran ggCaller on this isolate separately, along with its related reference genomes (see Table S1).

## RESULTS

### Survey of metadata

The historical strain collection at the IRLM contains 4,040 freeze-dried isolates. Based on the selection criteria, 3,331 isolates were excluded from this study. An additional 41 isolates were excluded due to duplication or missing ampule. Two isolates failed to amplify in the GenoType CM analysis and were also excluded, resulting in 666 historical isolates included in the study (comprising 24 with inferred GenoType CM results from a previous study).

For comparison with historical NTM isolates, data on 4,781 contemporary, NTM culture-positive isolates from any sample material received at the IRLM between 2013 and 2022 were extracted from the IRLM register at SSI. Excluded samples were those lacking successful species identification (*n* = 25), those originating from outside Denmark (i.e., Greenland, Faroe Islands, and Iceland; *n* = 45), or those representing follow-up samples from the same patient (*n* = 2,891), resulting in a final data set of 1,820 contemporary NTM isolates.

**Fig 1:**
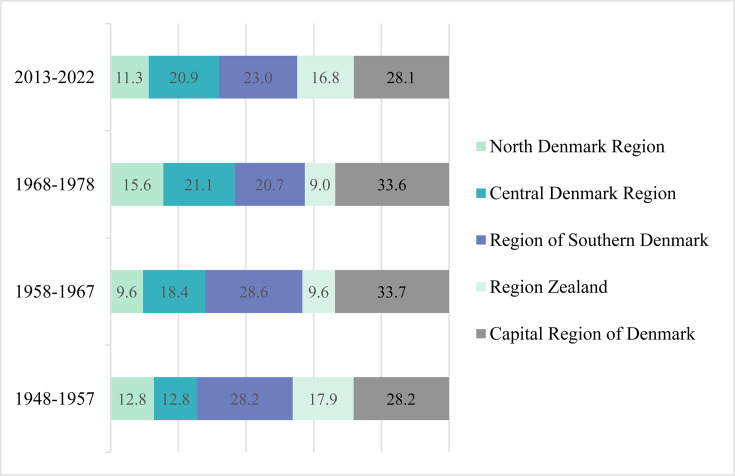
Geographical distribution of isolates across Danish regions, divided into approximate 10-year intervals. Historical samples refer to isolates collected between 1948 and 1978, and contemporary isolates refer to those collected between 2013 and 2022.

Analysis of available patient metadata showed median ages of 54.9 and 57.4 years for the historical and contemporary isolates, respectively. Most isolates in both data sets were from pulmonary-derived samples (89.1%; *n* = 596% and 78.9%, *n* = 1,436, respectively). Isolates from both periods also represented a broad geographical distribution across Denmark, with the highest proportion originating from the Capital Region (28.1%–33.7%; *n* = 512/1,820 [contemporary]–223/668 [historical]). The distribution of isolates across the five Danish regions was comparable over time (Fig. 1).

### Species determination of historical isolates using LPAs

An initial species determination of historical isolates was performed using GenoType CM, AS, and NTM-DR assays to identify the most common mycobacterial species encountered in clinical settings today. The GenoType CM analysis identified 16 distinct species categories (Table 1). The GenoType CM analysis designated unknown to 5 isolates due to unrecognized band patterns and failed to assign 214 (32.1%) of the NTM isolates to a known species, while it identified 14 distinct species among the remaining isolates (Table 1). The most frequently identified species were *M. gordonae* (18.6%, *n* = 124), *Mycobacterium avium* (17.0%, *n* = 113), and *M. intracellulare* (16.2%, *n* = 108). Additionally, frequently observed species included *Mycobacterium fortuitum* (4.2%, *n* = 28), *Mycobacterium malmoense* (3.3%, *n* = 22), and *Mycobacterium scrofulaceum* (2.0%, *n* = 13). In addition, two isolates were categorized as belonging to the MTBC based on GenoType CM, despite having previously been interpreted as NTM.

**TABLE 1:** GenoType results for CM, AS, and NTM-DR assay analyses of historical isolates (*n* = 666)

CM (*n*)	Band pattern	AS (*n*)	Band pattern	NTM-DR (*n*)
*M. avium* (113)	1–4			
*Mycobacterium* sp. (214)	1–3; 1–3,10			
*M. abscessus* (2)	1–3,5,6,10			*M. abscessus* subsp. *abscessus* (2)
*M. chelonae* (7)	1–3,5,10			
*M. fortuitum* (28)	1–3,7; 1–3,14; 1–3,7,14			
*M. interjectum* (1)	1–3,9–11			
*M. intracellulare* (108)	1–3,9			*M. intracellulare* (108)
*M. kansasii* (6)	1–3,10,12			
*M. malmoense* (22)	1–3,10,13; 1–3,9,10,13	*Mycobacterium* sp. (22)	1–3; 1–3,12	
*M. marinum/ulcerans* (1)	1–3,10,15	*Mycobacterium* sp. (1)	1–3,12	
*M. scrofulaceum* (13)	1–3,9,10			
*M. szulgai* (3)	1-3,10,11			
*M. xenopi* (17)	1–3,17; 1–3,10,17			
*M. gordonae* (124)	1–,8,10			
Unknown (5)	1–3,9,14; 1–2,5,10; 1–4,10; 1–4,8,10			
MTBC (2)	1–3,10,16			

Further analysis using AS for isolates classified as *M. malmoense* (*n* = 22) or *Mycobacterium marinum*/*ulcerans* (*n* = 1) revealed band patterns for *Mycobacterium* sp., supporting the identification as *M. malmoense* and *M. marinum*, respectively. Notably, only two isolates were identified as *M. abscessus* (0.3%) by GenoType CM and were subsequently confirmed as *M. abscessus* subsp. *abscessus* using NTM-DR.

### NTM clinical occurrence in Denmark over 70 years

To investigate long-term trends in NTM occurrence, species distribution from the historical data set was compared with contemporary routine diagnostic NTM data from IRLM, covering time frames of 30 and 10 years, respectively. Based on the distinct classifications obtained from the GenoType CM analysis, the single-species proportion was calculated and visualized.

**Fig 2:**
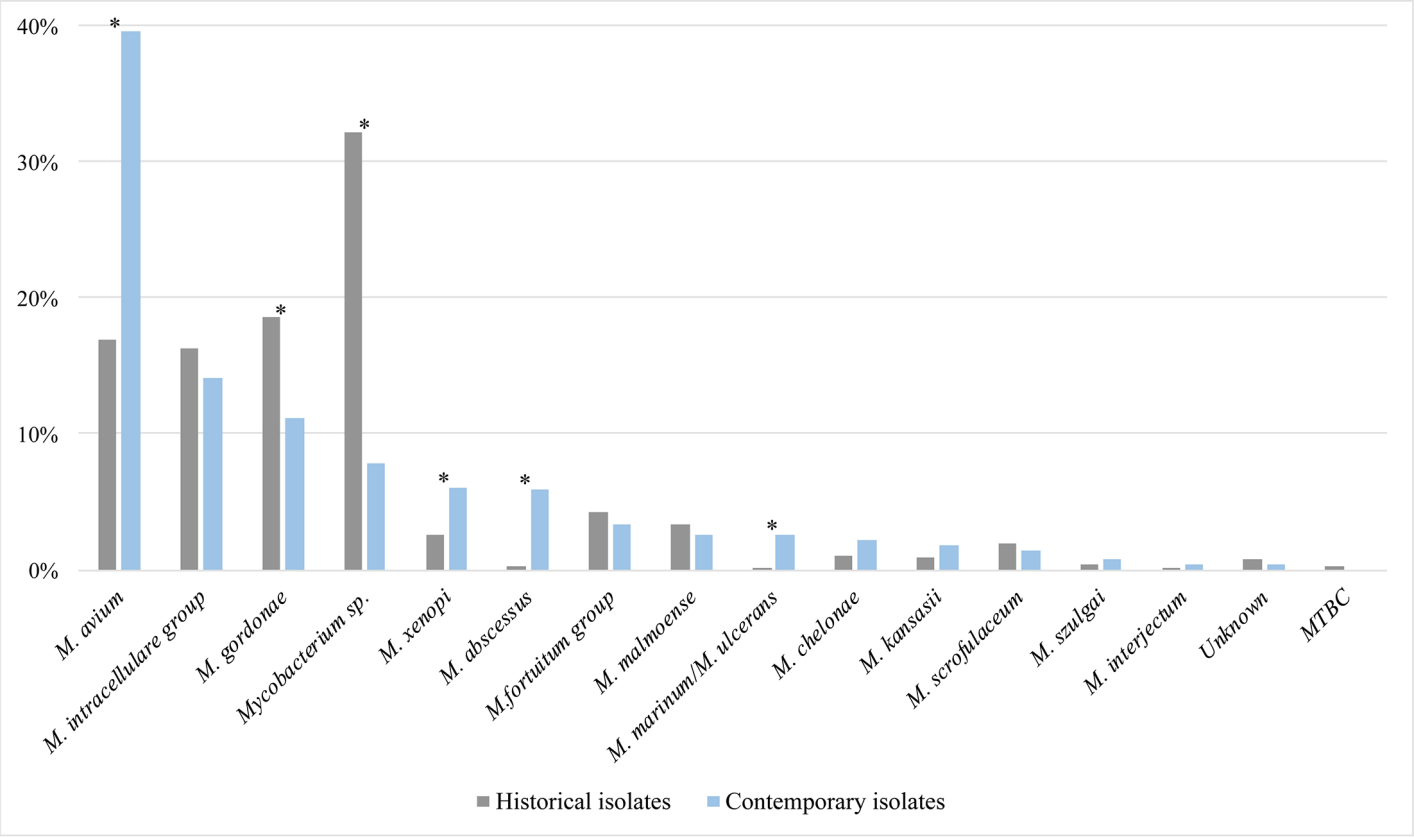
Proportion of single species identified in historical (*n* = 666) and contemporary (*n* = 1,820) isolate data sets, representing the periods 1948–1978 and 2013–2022, respectively. Species categories showing statistically significant changes in frequency between the two time periods are marked with * (Fisher’s exact test, *P* < 0.001).

The most frequently isolated species from 2013 to 2022 was *M. avium*, accounting for 39.6% of all NTM findings in the IRLM routine diagnostics, followed by the *M. intracellulare* (14.1%), *M. gordonae* (11.2%), and *Mycobacterium* sp. (7.8%) groups. Today, isolates initially classified as *Mycobacterium* sp. by GenoType CM undergo further species-level identification through additional analysis at IRLM. The same four taxa dominated the historical data set but in a different order: *Mycobacterium* sp., *M. gordonae*, *M. avium*, and the *M. intracellulare* groups. Six species categories showed significant changes in frequency between the two time periods (*P* < 0.001). The greatest increases were observed in *M. avium* and *M. abscessus*, whereas the *Mycobacterium* sp. category declined most markedly (Table 2; Fig. 2).

**TABLE 2:** Species identifications based on GenoType CM assay results, showing species-specific proportion within both historical and contemporary data sets

Species	Historical isolates (*n*)	Proportion	Contemporary isolates (*n*)	Proportion	Fisher’s exact test
*Mycobacterium* sp.	214	0.32	142	0.08	<0.001
*M. gordonae*	124	0.19	204	0.11	<0.001
*M. avium*	113	0.17	721	0.40	<0.001
*M. intracellulare*	108	0.16	256	0.14	0.179
*M. fortuitum*	28	0.04	61	0.03	0.330
*M. malmoense*	22	0.03	46	0.03	0.351
*M. xenopi*	17	0.03	110	0.06	<0.001
*M. scrofulaceum*	13	0.02	25	0.01	0.355
*M. chelonae*	7	0.01	39	0.02	0.092
*M. kansasii*	6	0.01	33	0.02	0.143
Unknown	5	0.01	6	0.00	0.177
*M. szulgai*	3	0.00	15	0.01	0.430
*M. abscessus*	2	0.00	108	0.06	<0.001
MTBC	2	0.00	0	0.00	0.072
*M. marinum/M. ulcerans*	1	0.00	46	0.03	<0.001
*M. interjectum*	1	0.00	8	0.00	0.459
Total	666	1.00	1,820	1.00	

### Species assignment and relatedness using WGS data

**Fig 3:**
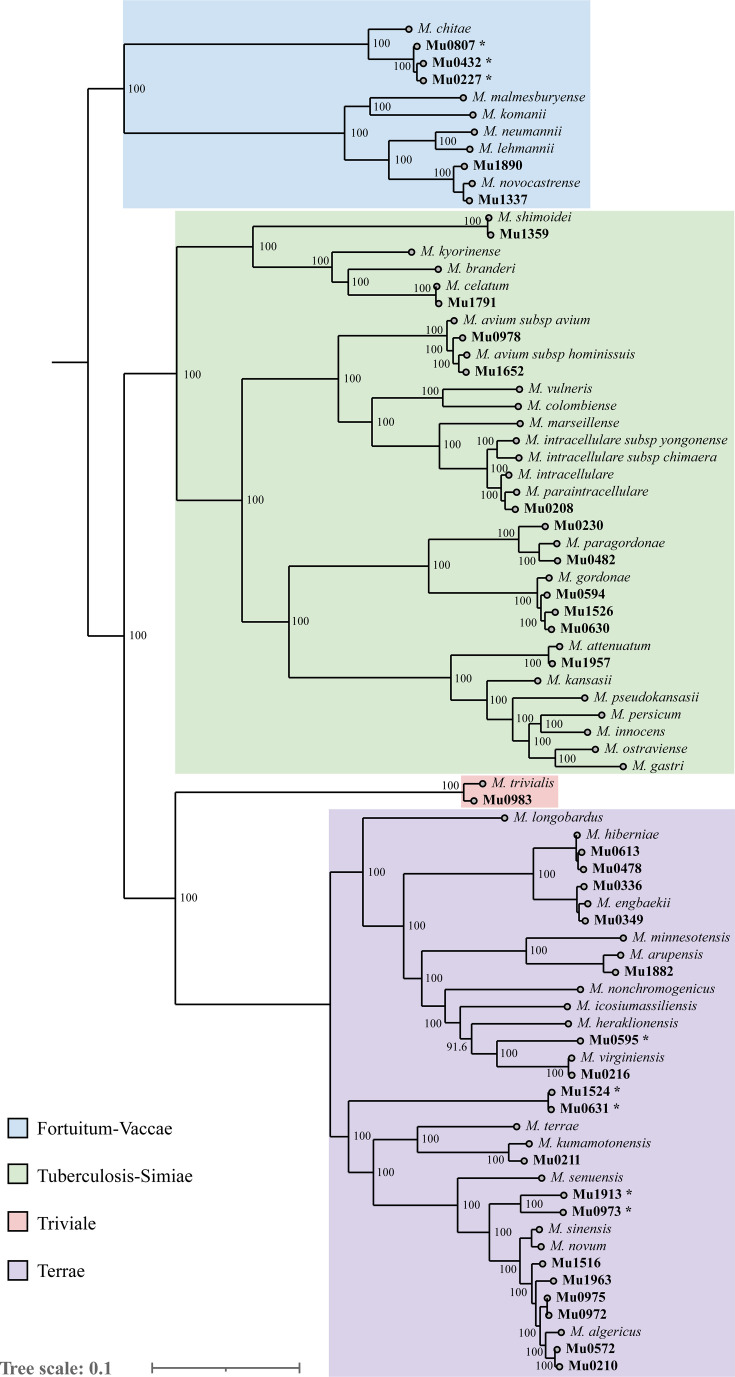
Phylogeny of 35 selected isolates based on core gene alignment obtained from ggCaller analysis, mapped to reference genomes from NCBI GenBank. Isolates with ANI scores <95% relative to the closest reference species are marked with *. Tree visualized using iTOL.

WGS-based species assignment was used to validate and further characterize isolates classified as *Mycobacterium* sp. (*n* = 30), *M. gordonae* (*n* = 5), or “unknown” (*n* = 5) (Table 3). Based on the constructed phylogeny, five isolates were excluded due to contamination with high numbers of nonmycobacterial (i.e., unclassified) reads: two “unknown” isolates (Mu0440 and Mu0851) and three *Mycobacterium* sp. isolates (Mu0433, Mu1365, and Mu1996) (Fig. 3).

**TABLE 3:** Species identification of 40 selected isolates based on WGS data using NTM-Profiler and FastANI^*a*^^,^^*b*^

Sample	GenoType CM classification	QC status	NTM-Profiler			FastANI	
			Species	ANI (%)	Abundance (× cov)	Species	ANI (%)
Mu0208	Unknown		*M. peregrinum + M. intracellulare*	>99	31	*M. intracellulare*	99.0
Mu0210	*Mycobacterium* sp.		*M. algericum*	98.9	36	*M. algericum*	98.5
Mu0211	*Mycobacterium* sp.		*M. kumamotonense*	98.71	22	*M. kumamotonense*	98.2
Mu0216	*Mycobacterium* sp.		*M. virginiense*	99.85	22	*M. virginiense*	99.9
Mu0227	*Mycobacterium* sp.	**	–		60	*M. chitae*	94.9
Mu0230	*M. gordonae*		*M. paragordonae*	97.47	27	*M. paragordonae*	96.8
Mu0336	*Mycobacterium* sp.		*M. engbaekii*	99.58	36	*M. engbaekii*	99.4
Mu0349	*Mycobacterium* sp.		*M. engbaekii*	99.6	38	*M. engbaekii*	99.3
Mu0432	*Mycobacterium* sp.	**	*–*		35	*M. chitae*	94.9
Mu0433	*Mycobacterium* sp.		*M. thermoresistibile*	98.76	3	*–*	
Mu0440	Unknown	Contaminated					
Mu0478	*Mycobacterium* sp.		*M. hiberniae*	99.83	77	*M. hiberniae*	99.9
Mu0482	*M. gordonae*		*M. paragordonae*	99.03	31	*M. paragordonae*	97.7
Mu0572	*Mycobacterium* sp.		*M. algericum*	98.92	55	*M. algericum*	98.5
Mu0594	*M. gordonae*		*–*		25	*M. gordonae*	98.8
Mu0595	*Mycobacterium* sp.	***	*–*		78	*M. virginiensis*	91.7
Mu0613	*Mycobacterium* sp.		*M. hiberniae*	99.72	61	*M. hiberniae*	99.7
Mu0630	*M. gordonae*		*–*		34	*M. gordonae*	98.8
Mu0631	*Mycobacterium* sp.	***	*–*		37	*M. senuensis*	85.7
Mu0807	*Mycobacterium* sp.	**	*–*		31	*M. chitae*	94.9
Mu0851	Unknown		*–*		44	*M. chelonae subsp. gwanakae*	98.5
Mu0972	*Mycobacterium* sp.		*M. algericum*	98.99	46	*M. algericum*	98.7
Mu0973	*Mycobacterium* sp.		*M. algericum*	99.08	26	*M. algericum*	95.8
Mu0975	*Mycobacterium* sp.		*M. algericum*	98.99	60	*M. algericum*	98.6
Mu0978	Unknown		*M. avium* + *M. engbaekii*	>99	35	*M. avium*	99.0
Mu0983	*Mycobacterium* sp.	*	*M. triviale*	99.05	64	*M. triviale*	98.6
Mu1337	*Mycobacterium* sp.		*M. novocastrense*	99.13	57	*M. novocastrense*	99.1
Mu1359	*Mycobacterium* sp.	**	*M. shimoidei*	99.38	34	*M. shimoidei*	99.7
Mu1365	*Mycobacterium* sp.		*–*			*–*	
Mu1516	*Mycobacterium* sp.		*M. algericum*	98.91	104	*M. algericum*	98.5
Mu1524	*Mycobacterium* sp.	***	*–*		65	*M. senuensis*	85.7
Mu1526	*M. gordonae*		*–*		18	*M. gordonae*	98.5
Mu1652	Unknown		*M. avium*	99.63	40	*M. avium subsp. hominisuis*	99.3
Mu1791	*Mycobacterium* sp.		*M. celatum*	99.97	52	*M. celatum*	100.0
Mu1882	*Mycobacterium* sp.	*	*M. arupense*	98.79	33	*M. arupense*	98.4
Mu1890	*Mycobacterium* sp.		*M. novocastrense*	98.99	51	*M. novocastrense*	98.7
Mu1913	*Mycobacterium* sp.	**	*M. algericum_A*	98.15	69	*M. novum*	94.6
Mu1957	*Mycobacterium* sp.		*M. attenuatum*	99.9	29	*M. attenuatum*	99.8
Mu1963	*Mycobacterium* sp.		*M. algericum*	99.03	45	*M. algericum*	98.5
Mu1996	*Mycobacterium* sp.	Contaminated	*M. attenuatum*	99.28	2	*–*	

^*a*^ Levels of unclassified reads are indicated as follows: *, low; **, medium; ***, high.

^*b*^ “–” indicates that the analysis did not find any match exceeding the ANI thresholds of 95% and 85% for NTM-Profiler and FastANI, respectively.

The core gene phylogeny showed that all 40 isolates were distributed across the distinct clades described by Gupta et al. (16) within the family *Mycobacteriaceae*. The phylogenetic clustering of isolates and reference genomes was consistent with WGS-assigned species, with only one exception: isolate Mu0208 showed closer proximity to *Mycobacterium paraintracellulare* but was assigned to *M. intracellulare* by ANI-based analysis (99.0%, Table 3).

Overall, results were consistent between contig-based (FastANI) and read-based (NTM-Profiler) species assignment, apart from mixed samples and isolates with only genus-level similarity to known type strains (85%–95%), where only FastANI reports matches. One isolate classified as unknown by LPA (Mu0208) was determined by NTM-Profiler to be a mixture of *Mycobacterium peregrinum* and *M. intracellulare*, while FastANI just assigned it to *M. intracellulare* (99.0%). Similarly, the unknown isolate Mu0978 was predicted to be a mixture of *M. avium* and *Mycobacterium engbaekii* by NTM-Profiler, while FastANI assigned it to *M. avium* (99.0%). The last unknown isolate (Mu1652) was assigned to *M. avium* subsp. *hominisuis* by FastANI (99.3%) but *M. avium* (99.6%) by NTM-Profiler, which currently does not resolve at the subspecies level (Table 3).

Species prediction of the isolates classified as *Mycobacterium* sp. by GenoType CM revealed several species rarely encountered in diagnostic settings today, such as *Mycobacterium algericum*, *Mycobacterium kumamotonense*, and *Mycobacterium hiberniae*. Isolates with only genus-level ANI scores (<95%) were clustered phylogenetically, with the reference species showing the highest ANI similarity, as observed for Mu0595 and *Mycobacterium virginiensis* (91.7%). Additionally, four isolates within the Terrae clade appeared to differ significantly from other known NTM species within the group. Isolates Mu1524 and Mu0631 were particularly distinct from their closest relative, *Mycobacterium senuensis*, with ANI scores as low as 85.7%. Two other isolates, Mu1913 and Mu0973, were identified as *Mycobacterium novum* and *M. algericum*, but with low ANI scores of 94.6% and 95.8%, respectively. The same tendency was observed for three isolates (Mu0807, Mu0432, and Mu0227; ANI: 94.9%), forming a separate cluster within the Fortuitum group, closest to the reference *Mycobacterium chitae*. In total, analysis of isolates assigned to *Mycobacterium* sp. revealed seven isolates with genus-level ANI scores, which were grouped into four distinct putative undescribed species. Additionally, a single isolate (Mu0973) exhibited an ANI score below 98% relative to *M. algericum*, indicating a putative undescribed subspecies.

Five isolates identified as *M. gordonae* by GenoType CM were selected for sequencing based on metadata to explore the potential for laboratory or hospital-related contamination. Two isolates (Mu0230 and Mu0482) were identified as *Mycobacterium paragordonae* by both NTM-Profiler and FastANI. These were collected in 1960 from the Region of Southern Denmark and the North Denmark Region, respectively. The other two isolates (Mu0594 and Mu0630) were collected in 1961 from the Region of Southern Denmark and the Central Denmark Region, while the final isolate, Mu1526, was collected in 1968 from a patient in the Capital Region of Denmark. All three isolates were identified as *M. gordonae* with FastANI (Table 3). The geographically widespread collection of *M. paragordonae* and *M. gordonae* isolates from patient sputum samples during the 1960s does not suggest intra-laboratory cross-contamination, which was further supported by the phylogeny, which showed a distinct separation between *M. paragordonae* and *M. gordonae* (Fig. 3).

## DISCUSSION

In this study, we present observations on the clinical occurrence of NTM in Denmark over the past seven decades by comparing contemporary trends with historical data. Species identification of 666 historical isolates suggested notable shifts in the mycobacterial species associated with human infections. Using GenoType CM, which is currently used in many laboratories for NTM diagnostics, 32.1% of the historical isolates were classified as *Mycobacterium* sp., reflecting unsuccessful specific species identification (17). This high proportion of unidentifiable species is striking, particularly compared to contemporary diagnostics, where only 7.8% of isolates received this designation between 2013 and 2022.

Further genomic analyses on a randomly selected subset of *Mycobacterium* sp.-classified historical isolates revealed species that are rarely identified in current clinical settings. These findings suggest a broader diversity of mycobacterial species associated with human infections in the mid- to late 1900s (Table 3). Interestingly, comparison of genomic similarity and relatedness to reference species in the NCBI RefSeq database showed that 7 of the 30 sequenced *Mycobacterium* sp. isolates from the historical data set clustered into four previously uncharacterized species, consistent with our previous identification of four new species in Iversen et al. (18) from the same collection. The small number of historical isolates contained a relatively large proportion of strains that could not be identified with GenoType CM. This suggests that people in the mid-20th century may have been exposed to and/or infected with a different spectrum of NTM than typically observed today. However, as only 30 isolates (14% of the 214 classified as *Mycobacterium* sp.) were sequenced, the findings may not fully capture the overall genetic diversity of NTM circulating during this period and may not be fully representative of the historical NTM population, despite their temporal distribution across years.

As seen in a recent study (13), the globally most prominent species in recent years—*M. avium* and *M. abscessus*—were also common in our contemporary data set. Similarly, this predominance was observed in two previous studies of NTM among Danish patients from 1991 to 2022 (19, 20). In contrast, only two historical isolates collected in 1957 and 1961 were identified as *M. abscessus*. This shift in NTM recovered from human samples may also partly reflect advances in diagnostic methodology, including improved culture media, the adoption of molecular species identification tools, and heightened clinical awareness of NTM disease (4).

A substantial number of historical isolates (19%, *n* = 124) were identified as *M. gordonae*, a species often linked to environmental or laboratory contamination since the late 20th century (21). Five GenoType CM classified *M. gordonae* isolates were further examined to assess the potential for contamination using a pan-genomic approach. Species assignment based on ANI calculations and phylogenetic analysis indicated that these isolates, collected across different years and Danish regions, were genetically distinct, making contamination from a single source unlikely. This historical prevalence of *M. gordonae*, relative to lower levels in the contemporary data set, may reflect differences in historical sampling practices, water handling, or laboratory processing. However, because *M. gordonae* is frequently associated with environmental contamination and detailed information on historical sampling and laboratory procedures is limited, this observation should be interpreted cautiously and may in part be influenced by methodological factors.

Multiple factors must be considered when investigating temporal trends in NTM occurrence, including clinical, social, and environmental variables. In recent decades, increasing recognition of NTM disease has likely been driven by an aging population, more widespread use of immunosuppressive therapies, and a higher burden of chronic diseases (13). Societal changes, such as urbanization and reduced exposure to rural environmental reservoirs such as agriculture, may also influence NTM exposure patterns and infection or disease risk.

A limitation of this study is the lack of documentation regarding the rationale for storing isolates in the historical collection. Nonetheless, we consider the strain collection to broadly represent clinically relevant mycobacterial infections during the period. All included isolates came from humans with suspected mycobacterial disease and were submitted by TB sanatoriums or hospitals, strongly suggesting the diagnostic relevance. However, it is unknown whether the isolates fulfilled the clinical microbiological criterion of three independent isolates required to distinguish environmental contamination from true infection, as later defined by the American Thoracic Society in 1997 (22). The high proportion of pulmonary samples (89.1%, *n* = 596) probably indicates clinical suspicion of pulmonary TB from 1948 to 1978 and supports the diagnostic value of the isolates included.

In conclusion, our findings have unveiled the spectrum of NTM species associated with human infections in Denmark over the past 70 years. Shifts in species prevalence may reflect a combination of diagnostic, societal, and environmental factors. Interestingly, sequencing of historical isolates unclassifiable by current routine diagnostic tools revealed several novel species and subspecies within the *Mycobacterium* genus, highlighting the value of archived collections in understanding long-term pathogen evolution.

## SUPPLEMENTAL MATERIAL

Figure S1 jcm.01561-25-s0001.pdf Flowchart illustrating the selection and exclusion of isolates from the historical strain collection, including applied inclusion and exclusion criteria.

Figure S2 jcm.01561-25-s0002.pdf Hain Lifescience Line Probe Assay classifications of the GenoType Mycobacterium CM v2.0 and AS v1.0 and GenoType NTM-DR v1.0 assays.

Figure S3 jcm.01561-25-s0003.pdf Phylogeny of isolate Mu0851 and its closest related reference genomes (NCBI GenBank) within the Abscessus-Chelonae clade.

Table S1 jcm.01561-25-s0004.xlsx NCBI genome assemblies of representative species and subspecies of mycobacteria included in ggCaller analyses.

ASM does not own the copyrights to Supplemental Material that may be linked to, or accessed through, an article. The authors have granted ASM a non-exclusive, world-wide license to publish the Supplemental Material files. Please contact the corresponding author directly for reuse.
