# Supplementary material for: Seven decades of nontuberculous mycobacteria in Denmark: shifts in species distribution and clinical relevance
Source: J Clin Microbiol. 2026 Apr 20;64(5):e01561-25. doi: 10.1128/jcm.01561-25 (PMC13170343; doi:10.1128/jcm.01561-25)
Supplement: Figure S1 — Flowchart illustrating the selection and exclusion of isolates from the historical strain collection, including applied inclusion and exclusion criteria. [file jcm.01561-25-s0001.pdf]

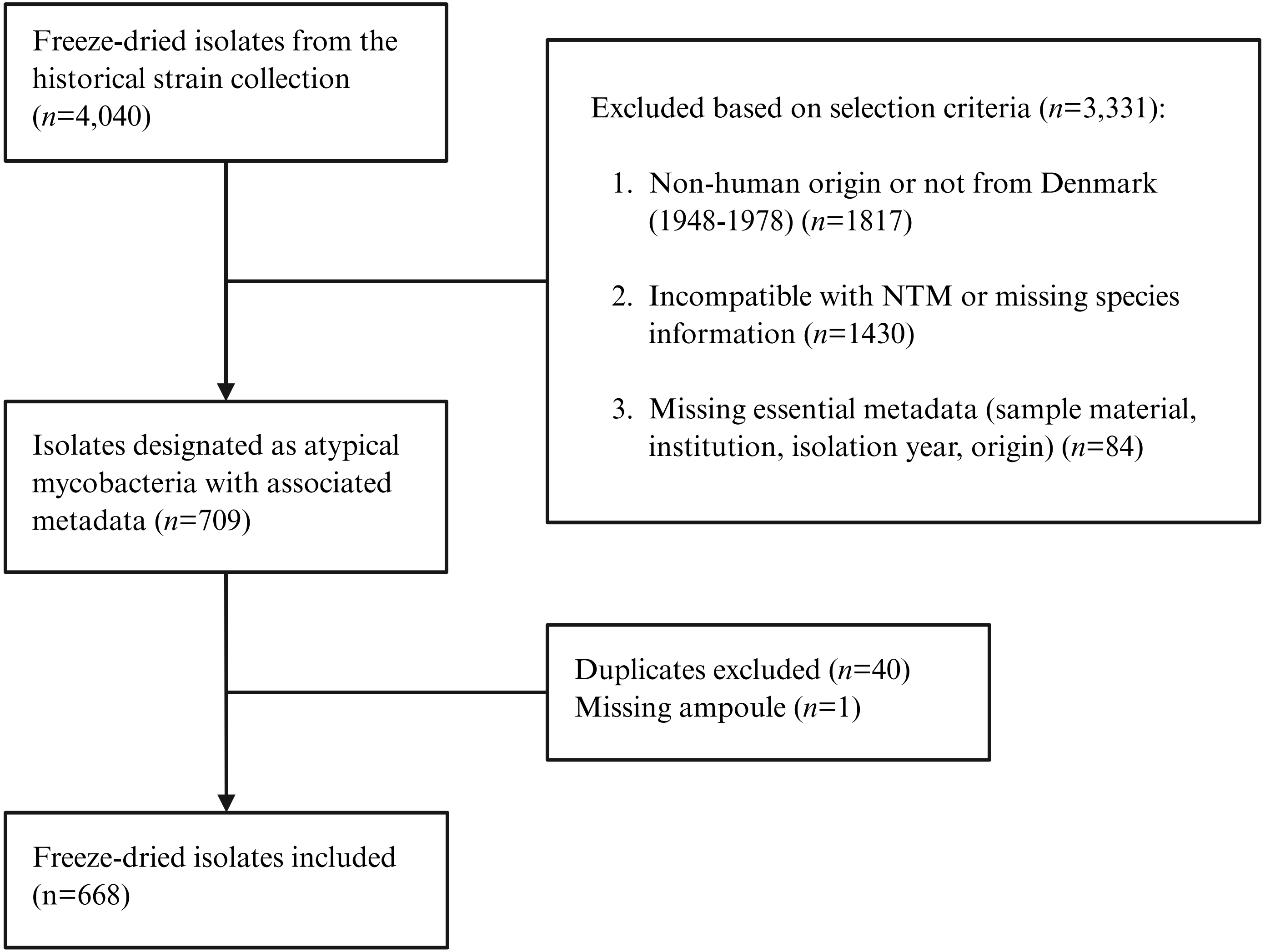

Figure S1: Flowchart illustrating the selection and exclusion of isolates from the historical strain collection, including applied inclusion and exclusion criteria. NTM; nontuberculous mycobacteria.
