# Supplementary material for: Seven decades of nontuberculous mycobacteria in Denmark: shifts in species distribution and clinical relevance
Source: J Clin Microbiol. 2026 Apr 20;64(5):e01561-25. doi: 10.1128/jcm.01561-25 (PMC13170343; doi:10.1128/jcm.01561-25)
Supplement: Figure S2 — Hain Lifescience Line Probe Assay classifications of the GenoType Mycobacterium CM v2.0 and AS v1.0 and GenoType NTM-DR v1.0 assays. [file jcm.01561-25-s0002.pdf]

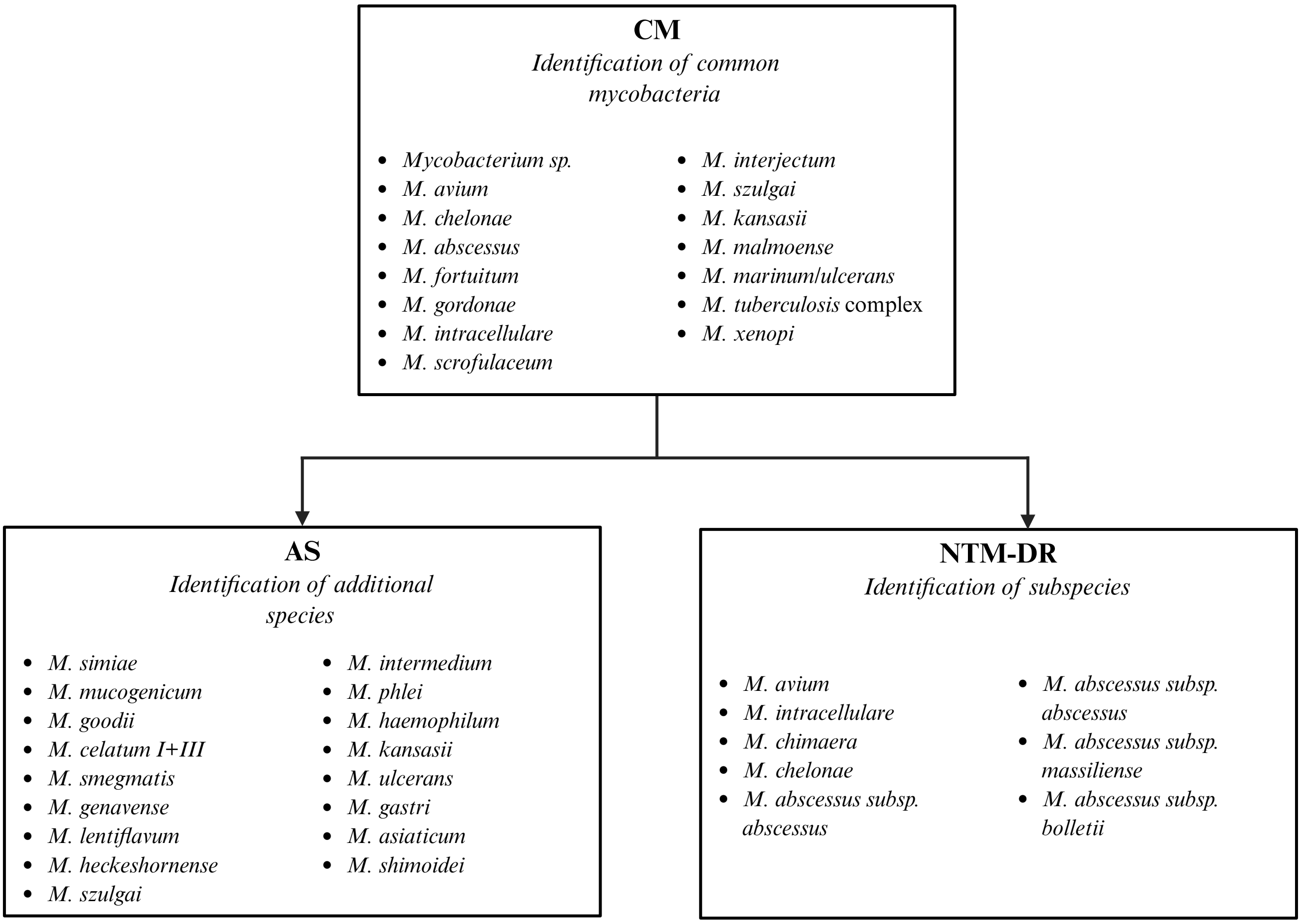

Figure S2: Hain Lifescience Line Probe Assay classifications of the GenoType Mycobacterium CM v2.0 and AS v1.0, and GenoType NTM-DR v1.0 assays.
