# Supplementary material for: Seven decades of nontuberculous mycobacteria in Denmark: shifts in species distribution and clinical relevance
Source: J Clin Microbiol. 2026 Apr 20;64(5):e01561-25. doi: 10.1128/jcm.01561-25 (PMC13170343; doi:10.1128/jcm.01561-25)
Supplement: Figure S3 — Phylogeny of isolate Mu0851 and its closest related reference genomes (NCBI GenBank) within the Abscessus-Chelonae clade. [file jcm.01561-25-s0003.pdf]

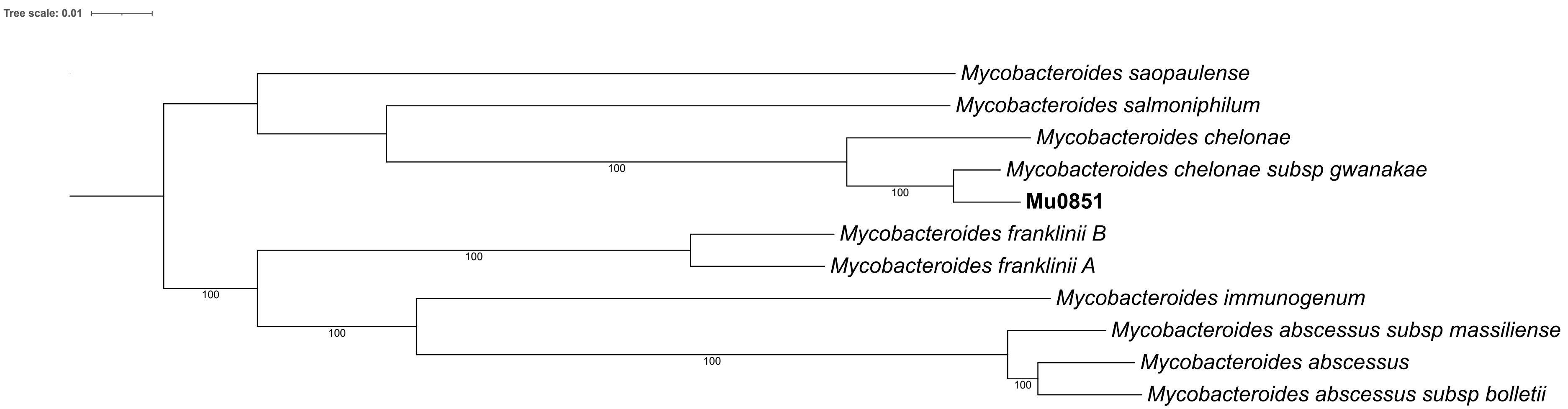

Figure S3: Phylogeny of isolate Mu0851 with closest related reference genomes (NCBI Genbank) of the Abscessus-Chelonae clade. The tree is based on core gene alignment obtained from ggCaller analysis and visualized using iTOL.
